# Curcumin as a Modulator of P-Glycoprotein in Cancer: Challenges and Perspectives

**DOI:** 10.3390/ph9040071

**Published:** 2016-11-10

**Authors:** Vanessa Lopes-Rodrigues, Emília Sousa, M. Helena Vasconcelos

**Affiliations:** 1 i3S, Instituto de Investigação e Inovação em Saúde, Universidade do Porto, Rua Alfredo Allen 208, 4200-135 Porto, Portugal; vrodrigues@ipatimup.pt; 2 Cancer Drug Resistance Group, IPATIMUP-Institute of Molecular Pathology and Immunology of the University of Porto, IPATIMUP, 4200-465 Porto, Portugal; 3 ICBAS-UP-Institute of Biomedical Sciences Abel Salazar, University of Porto, ICBAS-UP, 4099-003 Porto, Portugal; 4 Laboratory of Organic and Pharmaceutical Chemistry, Department of Chemical Sciences, Faculty of Pharmacy, University of Porto, Rua Jorge Viterbo Ferreira, 228, 4050-313 Porto, Portugal; esousa@ff.up.pt; 5 CIIMAR/CIMAR-Interdisciplinary Centre of Marine and Environmental Research, University of Porto, 4050-123 Porto, Portugal; 6 Laboratory of Microbiology, Department of Biological Sciences, FFUP-Faculty of Pharmacy, University of Porto, 4050-313 Porto, Portugal

**Keywords:** P-glycoprotein, multidrug resistance, curcumin, curcumin analogs

## Abstract

Multidrug resistance (MDR) presents a serious challenge to the efficiency of cancer treatment, and may be associated with the overexpression of drug efflux pumps. P-glycoprotein (P-gp) is a drug efflux pump often found overexpressed in cases of acquired MDR. Nevertheless, there are no P-gp inhibitors being used in the current clinical practice, due to toxicity problems, drug interactions, or pharmacokinetic issues. Therefore, it is important to identify novel inhibitors of P-gp activity or expression. Curcumin is a secondary metabolite isolated from the turmeric of *Curcuma longa* L. which has been associated with several biological activities, particularly P-gp modulatory activity (by inhibiting both P-gp function and expression). However, curcumin shows extensive metabolism and instability, which has justified the recent and intensive search for analogs of curcumin that maintain the P-gp modulatory activity but have enhanced stability. This review summarizes and compares the effects of curcumin and several curcumin analogs on P-glycoprotein function and expression, emphasizing the potential of these molecules for the possible development of safe and effective inhibitors of P-gp to overcome MDR in human cancer.

## 1. P-Glycoprotein as a Major Cause of Cancer Multidrug Resistance

Cancer cells may have inherent resistance or may develop resistance to antitumor drugs. In some cases, cells are cross-resistant to several unrelated (structurally and mechanistically different) drugs, a phenomenon known as multidrug resistance (MDR) [1,2].

**Figure 1:**
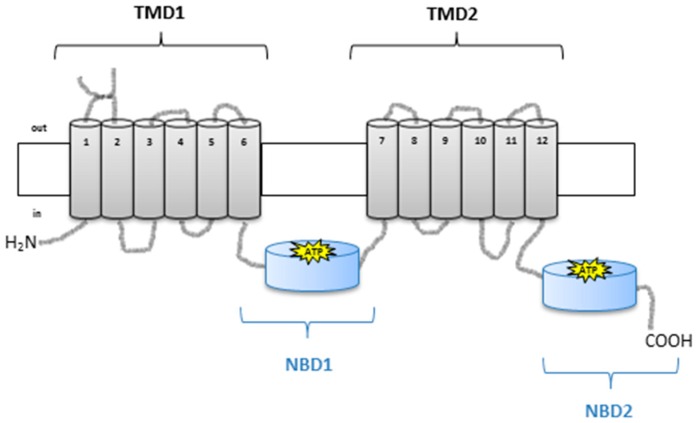
Schematic representation of P-glycoprotein (P-gp) structure with two halves, each with a transmembrane domain (TMD1 and TMD2) and a nucleotide-binding domain (NBD1 and NBD2) (adapted from [5]). The transmembrane domains (TMDs)—composed of six membrane α-helices (TM1–TM6 and TM7–TM12)—contain the drug binding sites and define the translocation pathway across the membrane; the two cytoplasmic nucleotide-binding domains (NBDs) couple the energy associated with ATP binding and hydrolysis, which is necessary for drug transport [6].

Overexpression of P-glycoprotein (P-gp) is one of the main mechanisms involved in the development of MDR [1,2]. P-gp (encoded by the *MDR1* gene, also referred to as ABCB1) is a drug-efflux pump from the ATP-binding cassette (ABC) transporters family, which efficiently removes cytotoxic drugs from the intracellular environment through an ATP-dependent mechanism [3]. This glycoprotein is composed of two highly hydrophobic integral membrane domains and two hydrophilic nucleotide-binding domains (Figure 1) [4].

Different studies have shown that by inhibiting P-gp, it is possible to counteract some cases of MDR in cancer [7]. There are many known MDR modulators belonging to several chemical classes, including calcium channel blockers, indole alkaloids, cyclosporines and calmodulin inhibitors [8]. However, the described modulators presented toxicity problems associated with the need for the use of very high doses in order to produce the required results [9]. To minimize these risks, new analogs of these compounds are being tested and developed, with the goal of finding potent MDR modulators with less toxicity problems [10].

Some of the most effective cancer treatments to date are derived from natural products, such as taxol [11]. Indeed, natural products and their synthetic derivatives comprise more than half of the approved anticancer drug candidates developed in the last decades [12]. Research on natural dietary phytochemicals from foods, herbs, and dietary supplements is increasing, due to the low expected toxicity.

The search for P-gp inhibitors has uncovered several natural compounds with this activity, such as flavonoids, quercetin, morin, catechins, capsaicin, and curcumin [13,14,15]. In this review, studies on the effects of curcumin and curcumin analogs on P-gp expression and activity are summarized, with the aim of highlighting the potential of these molecules for the possible development of safe and effective inhibitors of P-gp to overcome MDR in human cancer.

## 2. Curcumin as a Natural Product that Inhibits P-gp

**Figure 2:**
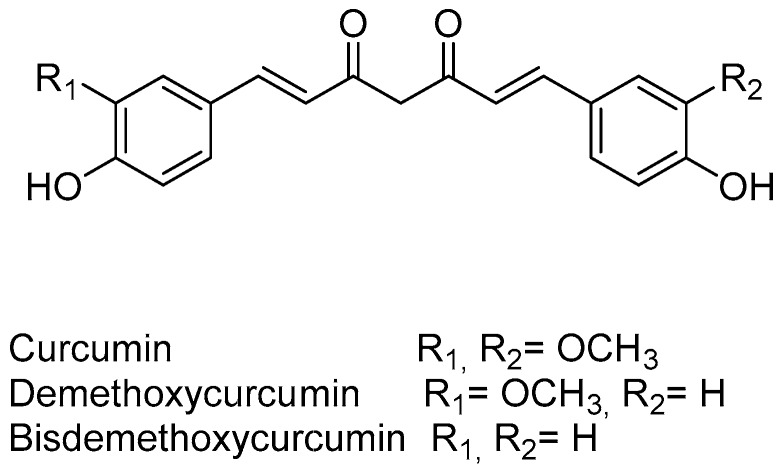
Main curcuminoids isolated from turmeric of *Curcuma longa* L.

Curcumin (Figure 2) is a phytochemical obtained from the dried rhizomes of *Curcuma longa* L. (turmeric), which has been used over the last centuries as a food additive, in cosmetics, and as a form of traditional herbal medicine [16]. It has a wide range of pharmacological activities, and presents low levels of toxicity.

Several studies have demonstrated a beneficial effect of curcumin in Alzheimer disease [17], and also in cases of high cholesterol through the inhibition of the intestinal absorption of cholesterol, consequently reducing its blood levels [18]. Curcumin was also described as an anti-HIV [19] and antibacterial agent [20]. Additionally, curcumin presents strong antioxidant, anti-inflammatory, and antiangiogenic properties, and was also described as having wound healing and anticancer effects [16,21].

Curcumin inhibits the growth of a wide variety of tumor cells, including gastric cancer [22], colon carcinoma [23], and breast cancer cells [24]. Additionally, over the years, extensive clinical trials have addressed curcumin’s capacity to treat patients with several types of cancer, such as colorectal cancer [25], pancreatic cancer [26], and prostate cancer [27]. Nevertheless, none of these clinical trials have reached phase III. Curcumin has been described as inhibiting the phosphorylation of protein kinase B (AKT )and AKT-related gene products, thereby presenting antitumor effects [28]. In addition, curcumin has been shown to downregulate AKT kinase signaling, inhibiting proliferation and inducing apoptosis in T-cell leukemia cell lines [29]. Interestingly, curcumin has also been described to be capable of suppressing P-gp expression and function, therefore reversing the MDR phenotype [30,31,32].

### 2.1. Curcumin as Modulator of P-Glycoprotein Function

Curcumin was described as having a role in reversing MDR through the modulation of P-gp. Specifically, curcumin has been described as an inhibitor of the function of P-gp in several in vitro and in vivo models [21,33].

Several studies revealed that the role of curcumin in the modulation of MDR occurred through its interaction with P-gp. In 2002, Anuchapreeda and collaborators [34] demonstrated that curcumin reduced P-gp-mediated drug-efflux in MDR cervical carcinoma cells in a dose-dependent manner. The authors also performed biochemical studies showing the direct interaction between P-gp and curcumin [34]. Two years later, the same group [35] purified the three major curcuminoids from the previously-used curcumin mixture [34] (curcumin, demethoxycurcumin, and bisdemethoxycurcumin, Figure 2) and analyzed the effect of the three curcuminoids on the modulation of P-gp function in the same MDR human cervical carcinoma cell line [35]. The authors suggested that these three curcuminoids are not substrates of P-gp, since they presented similar IC_50_ values for cytotoxicity between the MDR and the drug-sensitive cell lines used in the study [35]. In addition, they observed that curcumin was the most effective MDR modulator among the three curcuminoids, and could possibly be used in combination with conventional chemotherapy to reverse MDR in cancer [35].

Curcumin was also demonstrated to reverse MDR in a different model—a gastric carcinoma MDR cell line—possibly through a decrease in P-gp function which was observed in the treated cells [36]. In addition, two different MDR human cancer cell models (cervical and breast carcinoma MDR cell lines) that were treated with the three major curcuminoids (Figure 2) presented inhibition of P-gp function, which significantly increased the intracellular accumulation of rhodamine in a dose-dependent manner [37]. Additionally, the interaction of one of curcumin’s metabolites, tetrahydrocurcumin, with the P-gp molecule was clearly indicated by an ATPase assay and by photo affinity labeling of P-gp [37].

The above-mentioned studies were performed in order to study the capacity of curcumin to revert MDR. However, curcumin was also shown to have an effect in preventing the induction of MDR [38]. This was observed in a chronic myeloid leukemia cell line that was pre-treated with curcumin before being exposed for 24 h to doxorubicin with the purpose of selecting for a more resistant cell line (with overexpression of P-gp). These cells presented an increase in the intracellular rh123 accumulation levels, and an increase in the sensitivity towards doxorubicin (almost comparable to cells that were not exposed to doxorubicin), whereas the cells that were not pretreated with curcumin (but exposed to doxorubicin) presented a decrease in the intracellular rh123 accumulation levels and became more resistant to doxorubicin [38].

The previously mentioned studies reveal the potential of curcumin as an inhibitor of P-gp function. This particular activity of curcumin may contribute in the future to change clinical practices in cancer treatment, since the inhibition of P-gp function may help to counteract the MDR phenotype in some cancer patients.

### 2.2. Curcumin as Modulator of P-Glycoprotein Expression

Curcumin has been described to not only inhibit the function of P-gp, but also the expression of P-gp at the protein and at the mRNA level. This role of curcumin as inhibitor of the expression of P-gp was originally described in 2002 [34], in a work in which it was shown that treatment of MDR cervical carcinoma cells caused a decrease in the protein and mRNA levels of P-gp [34]. Later on, the same authors isolated three more important natural curcuminoids from turmeric (Figure 2, curcumin, demethoxycurcumin, and bisdemethoxycurcumin) and compared them for their ability to modulate P-gp expression in the same model (a MDR human cervical carcinoma cell line) [39]. They have demonstrated that bisdemethoxycurcumin was the most active of the curcuminoids present in turmeric regarding the inhibition of the P-gp expression. In addition, treatment of the MDR cells with curcumin increased their sensitivity to vinblastine, which was consistent with a decrease in P-gp expression [39].

The effect of curcumin in P-gp expression was also demonstrated in other MDR cancer cell models, such as in a human MDR gastric cancer cell line [36], a human MDR colon cancer cell line [40], and a mouse MDR leukemia cell line [31], in which it was described that the inhibitory effect of curcumin in the expression of P-gp ultimately led to MDR modulation and cellular sensitization to common drugs. From a mechanistic point of view, it was proposed that curcumin can contribute to counteract the MDR phenotype, probably by suppression of P-gp expression via inhibition of the Phosphatidylinositol-4,5-bisphosphate 3-kinase/AKt/factor nuclear kappa B (PI3K/Akt/NF-kB) signaling pathway [31].

The role of curcumin in preventing MDR by decreasing P-gp expression in a chronic myeloid leukemia model was also shown [38]. In addition, it was found that all the three curcuminoids (curcumin, demethoxycurcumin, and bisdemethoxycurcumin, Figure 2) were capable of preventing MDR induced by doxorubicin in that model (chronic myeloid leukemia). However, the preventive effect of each curcuminoid was different, with demethoxycurcumin being the most active of the curcuminoids for the prevention of *MDR1* mRNA and P-gp overexpression [41].

Finally, it was demonstrated that both the chemotherapeutic and chemosensitizing effects of curcuminoids (by downregulation of P-gp expression) in colon cancer cells were mediated through modulation of a microRNA (miR-27a-ZBTB10-Sp-axis) [42].

Although these works have demonstrated that curcumin is an effective inhibitor of P-gp expression in vitro, animal experimentation was further required to determine if curcumin had potential as an effective and safe chemosensitizer. Curcumin was shown to have the capacity to inhibit the expression of P-gp in xenografts of human colon cancer cells in mice. Indeed, curcumin treatment significantly reduced the expression of the MDR1 gene and of P-gp protein in the xenografts of mice, when compared to the control group [40]. These results suggested that curcumin could partially reverse the MDR phenotype. Additionally, curcumin inhibited xenograft growth [40]. The P-gp inhibitory activity of curcumin was also demonstrated in an in vivo model of colon cancer, by a novel method—the in situ cancerous colonic single pass perfusion method in rats [32].

The above-mentioned role of curcumin as inhibitor of P-gp expression in different in vitro and in vivo cancer models further strengthens the need for more clinical trials to study the clinical feasibility of using curcumin to counteract the MDR phenotype in cancer patients.

## 3. Strategies for Overcoming the Limitations of Curcumin

The previously mentioned studies suggested that curcumin could become a promising lead compound for overcoming MDR in cancer. However, this compound is very unstable, and has low bioavailability in vivo [21]. Indeed, curcumin is highly hydrophobic, which prevents its absorption across the gut, therefore having a rapid metabolism and a limited tissue distribution [43]. In summary, the instability of the molecule and its poor pharmacokinetics profile are major drawbacks for its possible clinical use. This became evident in a Phase I clinical trial, during which very high oral doses of curcumin (8–12 g/daily) resulted in peak plasma concentrations in the nanomolar range only [44,45]. Therefore, two strategies have been employed in order to overcome this problem: the incorporation of curcumin into pharmaceutical formulations to improve its delivery, and the design and synthesis of analogs of curcumin aiming to obtain better stability and bioavailability.

### 3.1. Improving the Intracellular Delivery of Curcumin

The delivery of curcumin may be improved with the help of nanotechnology. Indeed, it has been reported that a more pronounced downregulation of P-gp expression was observed when curcumin was delivered in nanoemulsion formulations (heterogeneous mixtures of oil in water, where the oil droplets have a nanometer size, which allow the solubilization of curcumin, since it is a hydrophobic compound) when compared to curcumin alone [46]. In this study, human MDR ovarian adenocarcinoma cells were treated with paclitaxel and curcumin, both encapsulated in nanoemulsion formulations. The results obtained showed that the encapsulated drugs were effectively delivered, and that curcumin downregulated P-gp expression in a more effective way than when administered without being encapsulated in nanoemulsions [46]. Another study used lipid nanoparticles loaded with doxorubicin and curcumin to treat mice with induced hepatocellular carcinoma. This combined formulation increased cellular cytotoxicity and decreased P-gp expression [47].

It is also possible to use polymers to improve the delivery of curcumin. Indeed, in a recent study, MDR breast cancer cells were treated with a biotin–Polyethylene glycol (PEG)–PCDA (poly(curcumin-dithio dipropionic acid) polymeric nanocarrier loaded with paclitaxel, magnetic nanoparticles, and quantum dots [48]. The nanoparticles presented high uptake by tumor cells, and curcumin (cleaved product of PCDA) downregulated the expression of P-gp, allowing an increase in paclitaxel accumulation within the target cancer cells, thereby enhancing the therapeutic efficacy against the MDR cells [48]. Furthermore, it was shown that curcumin encapsulated in chitosan–polybutyl cyanoacrylate nanoparticles decreased the expression levels of P-gp in MDR cells when compared to control treatments with curcumin alone [49]. In this study, MCF-7-doxorubicin-resistant cells were treated with several preparations of chitosan–polybutyl cyanoacrylate nanoparticles containing curcumin, doxorubicin, or both, and the cellular P-gp expression levels and the cytotoxic effects were then assessed. The results showed a significant improvement in the cytotoxic effect and in the P-gp inhibitory effect when using encapsulated curcumin [49]. Moreover, polylactic-co-glycolic acid nanoparticles containing curcumin were described in another study as having a higher cellular uptake and cytotoxic effect in cervical cancer cells, when compared to curcumin alone [50]. Furthermore, in a subsequent study, these authors conjugated the same nanoparticles (loaded with curcumin) with an anti-P-gp antibody in order to obtain a more targeted therapy [51]. These new nanoparticles presented a more pronounced cytotoxic effect against an MDR cervical cancer model than the nanoparticles without the P-gp antibody [51].

The encapsulation of curcumin (alone or in combination with another drug) within micelles has also been described [52,53,54,55]. The improved effects of this type of formulation could be attributed to the solubilization of the hydrophobic curcumin in the micelle core, together with the P-gp inhibitory effect of curcumin. For instance, a preparation of mixed micelles (polymeric micelles and surfactant micelles) loaded with curcumin caused a significant improvement in the cytotoxic activity on human MDR lung cancer cells and in their oral bioavailability, when compared to curcumin alone [52]. In addition, a Poloxamer 407 and a D-alpha-tocopheryl polyethylene glycol 1000 succinate mixed micelle with encapsulated curcumin, were developed and tested in MDR ovarian cancer cells [53]. The micelles containing curcumin were capable of inducing an increase in the intracellular uptake of curcumin, as well as an increase in the cytotoxic effect (when compared to curcumin alone) [53]. Another micelle preparation (pegylated polymeric micelle) was developed to co-encapsulate doxorubicin and curcumin [54]. The co-delivered micelles exhibited excellent cytotoxicity, reverted the MDR phenotype, and promoted an increase in the cellular uptake of doxorubicin, which induced apoptosis in MCF7/Adr MDR cells [54]. Finally, in another work, amphiphilic polymeric micelles were prepared, also in order to encapsulate doxorubicin and curcumin [55]. MCF7/Adr MDR cells were treated with those micelles, and the cellular uptake of doxorubicin, the cellular drug efflux rate, the downregulation of P-gp expression, and the inhibition of ATP activity were assessed. Results showed a higher cellular uptake and lower efflux rate of doxorubicin, and a stronger effect on the downregulation of P-gp and inhibition of ATP activity in cells treated with the co-encapsulated micelles, when compared to control treatments (curcumin and/or doxorubicin alone). Additionally, those micelles presented increased tumor accumulation and stronger inhibitory effect on tumor growth in a xenograft model of the same MDR cells, when compared to other drug formulations [55].

All of the above-mentioned strategies using pharmaceutical formulations to improve curcumin delivery proved to be important approaches to overcome the low bioavailability of curcumin.

### 3.2. Curcumin Derivatives and Analogs as Inhibitors of P-gp

Over the past few years, several curcumin derivatives/analogs have been synthesized with the objective of obtaining molecules more stable and bioavailable than curcumin, while not losing their biological activity (Table 1).

Several authors have attempted to understand the MDR modulatory effect of curcumin and the disadvantages that curcumin presents in terms of pharmacokinetics profile from a chemical perspective. They have attempted to synthesize better compounds; i.e., maintaining the MDR modulatory effect while improving the stability and availability of curcumin.

Given the importance of curcumin in cancer treatment, several curcumin derivatives/analogs have been synthesized and studied over the past few years; however, only a few candidates have been studied as MDR modulators (by inhibiting P-gp function and/or P-gp expression). For instance, 12 unsymmetrical curcuminoids with various amide groups were synthesized and tested for MDR reversal activity. These compounds were tested in MDR cervical adenocarcinoma P-gp overexpressing cells, together with vincristine and paclitaxel (in comparison with verapamil as a positive control), in order to access their MDR modulatory capacity. Three of the compounds presented a potent MDR reversal activity by inhibiting the drug efflux function of P-gp, while the others were only moderately potent [56]. This work also showed—from the preliminary structure–activity study—that only half of the curcumin symmetrical structure is a promising lead structure for a MDR reversal agent, particularly one or two chloride groups at the *meta*- or *para*- position on benzamide [56].

Moreover, monocarbonyl analogs of curcumin have been described to be more stable than curcumin, since they do not have the unstable β-diketone moiety of curcumin [60]. Nonetheless, most studies with monocarbonyl analogs have focused on the anticancer, antibacterial, anti-inflammatory, and antioxidant effects, and very little is known about their ability to inhibit ABC transporters such as P-gp. Indeed, 23 heterocyclic cyclohexanone monocarbonyl analogs of curcumin were synthesized, and were initially investigated for their possible inhibitory effects on the function of several ABC transporters, including P-gp (using flow cytometry and resistance reversal assays) [57]. Besides being more stable than curcumin, many of these compounds inhibited P-gp function, most of them being more potent than curcumin itself. Moreover, some of these analogs showed potent anticancer activity [57], which raised the possibility of using them as antitumor dual agents (similarly to the above described curcumin effect).

Additionally, the cytotoxic and P-gp inhibitory activities of 19 chloro and asymmetrical series of synthetic curcumin derivatives were studied in a MDR acute lymphoblastic leukemia model, and in its drug-sensitive counterpart cell line [58]. Four moieties have been explored for structural modification of curcumin: the aryl side chain, the diketo group, the double bond, and the active methylene groups. Based on these hypotheses, 19 new compounds were synthesized, and the biological results obtained showed that MDR cells exhibited cross-resistance to 10 compounds, collateral sensitivity to three compounds, and regular sensitivity to the other six compounds. Some of them caused a significant increase in doxorubicin uptake rates (an effect that was better than that observed with verapamil), indicating a remarkable inhibition of P-gp function (since doxorubicin is a P-gp substrate) [58]. However, in terms of ATPase activity, results showed that the novel compounds did not induce basal (and verapamil-stimulated) P-gp-ATPase activity in a comparable manner to verapamil [58].

Finally, five diketone and four cyclohexanone curcumin analogs were synthesized, and the modified substitution moieties were on the aromatic ring, while four of the cyclohexanone analogs were investigated by replacing the di-ketone with cyclohexanone having modifications on the functional groups on the aromatic ring. These compounds were analyzed regarding the intracellular accumulation of P-gp substrates (such as rhodamine 123 and doxorubicin) in a MDR chronic myeloid leukemia model, in order to evaluate their effect as inhibitors of P-gp function. Two of these compounds (1,7-bis-(3,4-dimethoxy-phenyl)-hepta-1,6-diene-3,5-dione and 2,6-bis-(3,4-dimethoxy-benzylidene)-cyclo-hexanone) caused a strong inhibitory effect of P-gp function [59], which suggests that these analogs may be strong MDR modulators.

From the above-mentioned published studies, only one study investigated the inhibitory activity of the analogs in terms of P-gp expression [59]. From the studied diketone and four cyclohexanone curcumin analogs mentioned above, three of them showed a strong effect as inhibitors of P-gp expression. Interestingly, one of the analogs (2,6-bis-(3,4-dimethoxy-benzylidene)-cyclo-hexanone) caused simultaneous inhibition of the expression and function of P-gp [59]. This compound might be a very good candidate for an MDR modulator, since it has dual inhibitory effects (on both P-gp function and expression), increased the sensitivity of the MDR cells to paclitaxel, and also has better stability than curcumin itself.

These studies provide a basis for further systematic activity improvement of synthetic curcumin derivatives and analogs. Curcumin exerts MDR reversal activity, and therefore chemical modifications of curcumin may improve its biological activities. Therefore, curcumin derivatives and analogs should be investigated in more detail, in order to address the limitations of curcumin and to improve its biological activities—in particular the P-gp modulatory effect, which is still not very thoroughly explored. Moreover, it will be important to improve the dual activity of curcumin derivatives or analogs, since stable concomitant inhibitors of tumor growth and P-gp activity are promising leading compounds to proceed to clinical studies in order to improve treatment of MDR cancer patients.

## 4. Future Perspectives

It is believed that the level of P-gp in cell lines is much higher than that in human tissues [61], which means that lower concentrations of P-gp modulators should be effective inhibitors of P-gp in vivo, when compared to in vitro studies. Therefore, even though curcumin and derivatives are considered to be non-toxic, a low therapeutic dose could be favorable in future in vivo studies.

Future studies should also consider the synergistic effects of curcumin with other compounds, on P-gp transporter function and expression. Combinatorial studies with several natural compounds or with conventional anticancer drugs could be carried out in order to identify potential synergistic or additive effects.

## Figures and Tables

**Table 1:** Reported curcumin derivatives/analogs with P-gp modulatory effect.

Compounds	MDR Cancer Model	Inhibitors of P-gp Function	Inhibitors of P-gp Expression	References
Unsymmetrical curcumin mimics with various amide moieties	MDR cervical adenocarcinoma	X		[56]
Heterocyclic cyclohexanone monocarbonyl analogs of curcumin	Human embryonic kidney cells and canine kidney cells transfected with wild-type P-gp	X		[57]
Chloro and asymmetrical series of synthetic curcumin derivatives	MDR acute lymphoblastic leukemia	X		[58]
Diketone and cyclohexanone curcumin analogs	MDR chronic myeloid leukemia	X	X	[59]

MDR: multidrug resistant.
